# Clinical Significance of HSCARG for Atherosclerotic Coronary Heart Disease and Reduced ROS-Oxidative Stress in *in Vivo* and *in Vitro* Models via p47phox by NF-κB Activity

**DOI:** 10.21470/1678-9741-2021-0183

**Published:** 2022

**Authors:** Xiaofang Zhou, Siwei Zhou, Yuanmei Li, Zhiyong Qian, Chao Zeng, Yang Li

**Affiliations:** 1 Geriatric Rehabilitation Center, Zhejiang Rehabilitation Medical Center, Hangzhou, Zhejiang, People’s Republic of China.

**Keywords:** NF-kappa B, Reactive Oxygen Species, Oxidative Stress, Angiotensin II, Human Umbilical Vein Endothelial Cells, Coronary Diseases

## Abstract

**Introduction:**

Coronary heart disease (CHD) is a dynamic process in which there are interactions between endothelial dysfunction, oxidative stress, and inflammatory responses. The aim of the present study was to investigate the function and mechanism of HSCARG in the treatment of CHD.

**Methods:**

Male apolipoprotein E/low-density lipoprotein receptor-deficient mice were given a high-fat diet with 21% fat and 0.15% cholesterol for the in vivo model. Human umbilical vein endothelial cells were incubated with angiotensin II for the in vitro model. HSCARG expression was inhibited in patients or mice with CHD.

**Results:**

HSCARG reduced oxidative stress in mice with CHD. HSCARG also reduced reactive oxygen species (ROS)-oxidative stress in the in vitro model. HSCARG induced p47phox expression in the in vitro model by NF-κB activity. The regulation of nuclear factor kappa B (NF-κB) activity or p47phox expression participates in the effects of HSCARG in CHD.

**Conclusion:**

Altogether, our data indicate that HSCARG reduced ROS-oxidative stress in in vivo and in vitro models of CHD via p47phox by NF-κB activity and may be a clinical target for CHD.

**Table:** Abbreviations, Acronyms & Symbols

ApoE/LDLR-/-	= Apolipoprotein E/low-density lipoprotein receptor-deficient	LVEF	= Left ventricular ejection fraction
AS	= Atherosclerosis	LVESd	= Left ventricular end-systolic diameter
AUC	= Area under the curve	MDA	= Malondialdehyde
BUN	= Blood urea nitrogen	mRNA	= Messenger ribonucleic acid
CHD	= Coronary heart disease	NADPH	= Nicotinamide adenine dinucleotide phosphate
DAPI	= 2-(4-Amidinophenyl)-6-indolecarbamidine dihydrochloride	NF-κB	= Nuclear factor kappa B
DNA	= Deoxyribonucleic acid	NOX	= NADPH oxidase
EF	= Ejection fraction	PCR	= Polymerase chain reaction
ELISA	= Enzyme-linked immunoassay	RIPA	= Radioimmunoprecipitation assay buffer
FC	= Fold change	RNA	= Ribonucleic acid
FS	= Fractional shortening	ROS	= Reactive oxygen species
GSH	= Glutathione	SCr	= Serum creatinine
GSH-PX	= Glutathione peroxidase	SOD	= Superoxide dismutase
LVEDd	= Left ventricular end-diastolic diameter		

## INTRODUCTION

Atherosclerosis (AS) is an arterial vascular disease characterized by vascular sclerosis and lumen stenosis caused by lipid metabolism disorder and abnormal regulation of inflammatory response, which is the main cause of atherosclerotic coronary heart disease (CHD)^[1]^. Vascular endothelial cell damage is the central link in the occurrence and development of AS, therefore, markers related to vascular endothelial damage may have diagnostic value for CHD^[2,3]^.

With the social and economic development, the prevalence of risk factors of cardiovascular disease has been significantly increasing^[4]^. Also, the number of deaths caused by CHD is rising^[5]^. The death rate of CHD was close to 1/1000 by 2012, which is the first cause of death for Chinese residents^[6]^. The treatment of CHD includes not only the active vascular reconstruction after the onset, but more importantly, full management and secondary prevention^[6]^. Studies have shown that the 10-year risk of death after percutaneous coronary intervention is still over 30%; 32.3% of patients have experienced angina in the first year; and the rate of in-stent restenosis is 1%. Meanwhile, patients have high-risk recurrent events and bear a greater financial burden^[6]^.

Recent studies have shown that, in addition to direct cytotoxic effects, oxidative stress can also regulate the expression of certain genes by regulating the cellular signal transduction system^[7-9]^. The selective expression of inflammation-related genes induced by intracellular oxidative stress signals may be the common molecular mechanism that triggers plaque formation and development of CHD^[9]^.

Nicotinamide adenine dinucleotide phosphate (NADPH) oxidase, also known as NADPH oxidase (NOX), family contains different nitrogen oxide subunits (NOX 1-5, DUOX1, DUOX2) which are the main sources of expression of reactive oxygen species (ROS) in non-phagocytic cells. A variety of enzymes are involved in the production of reactive oxygen in the body, such as NOX, mitochondrial respiratory chain complex enzyme, xanthine oxidase, cytochrome P450, nitric oxide synthase, etc^[10]^. Among them, NOX is an important source of reactive oxygen in the body. And p47phox plays a crucial role in activating NOX^[11]^.

The human protein HSCARG, also known as NMRAL1 (or nitrogen metabolite repression regulator-like family domain-containing protein 1), has been identified as a NADPH sensor. Histone ubiquitination plays an important role in many aspects of deoxyribonucleic acid repair and transcription regulation. HSCARG interacts with polycomb repressive complex (or PRC1) and ubiquitin specific protease 7 (or USP7) to inhibit ubiquitination^[12]^. Knockout of HSCARG can cause the continuous activation of cell cycle checkpoint signals, thereby causing cell cycle arrest^[13,14]^. Zang et al.^[14]^ expanded that HSCARG negatively regulates the translesion synthesis pathway and reduced oxidative stress. So, we thought HSCARG may reduce oxidative stress in CHD, and the aim of the present study was to investigate the function and mechanism of HSCARG in the treatment of CHD.

## METHODS

### Clinical Trial

The experiments in the present study were approved by the Ethics Committee of Zhejiang Rehabilitation Medical Center (Nº 195871). Patients with CHD and normal healthy volunteers were gathered from the Zhejiang Rehabilitation Medical Center. Serum samples of patients with CHD and normal healthy volunteers were collected and saved at-80 ºC.

CHD patients were diagnosed with angiographic evidence of at least one segment of a major coronary artery, including the left anterior descending, left circumflex, or right coronary artery, with > 50% organic stenosis. The patients were included in this study if they had no family history of CHD and no history of significant concomitant diseases, including hepatic failure, renal failure, hepatitis, cardiomyopathy, congenital heart disease, bleeding disorders, previous thoracic irradiation therapy, and malignant diseases.

### Animals Model and Grouping

Male apolipoprotein E/low-density lipoprotein receptor-deficient (ApoE/LDLR-/-) mice (4-5 weeks, 18-20 g) were randomly assigned into two groups: control group (normal diet group, n=8) and CHD group (high-fat diet group, n=8). All mice were housed under specific pathogen-free conditions at a temperature of 22 °C and 12-hour dark/light cycle and allowed standard rodent diet and water *ad libitum*. CHD mice were given a high-fat diet with 21% fat and 0.15% cholesterol for 16 weeks as references^[1]^ Control mice were given normal saline for 16 weeks. All procedures were approved by the Animal Ethics Committee of Zhejiang Rehabilitation Medical Center. Next, male ApoE/LDLR-/- mice (4-5 weeks, 18-20 g) were randomly assigned into two groups: CHD group (normal diet group, n=8) and CHD+HSCARG group (high-fat diet group, n=8). CHD mice were given a high-fat diet with 21% fat and 0.15% cholesterol for 16 weeks. CHD+HSCARG mice were given a high-fat diet with 21% fat and 0.15% cholesterol for 16 weeks and treated with human HSCARG protein (1 µg/day/week) for 16 weeks.

### Assessment of Cardiac Functions

Mice were anaesthetized and cardiac functions were measured: left ventricular end-systolic diameter (LVESd), left ventricular end-diastolic diameter (LVEDd), ejection fraction (EF), and fractional shortening (FS). EF = [(left ventricular end-diastolic volume - left ventricular end-systolic volume)/left ventricular end-diastolic volume] × 100%; and FS was calculated using the equation: [(LVEDd - LVESd)/LVEDd] × 100%. Ultrasound was performed to survey and calculate left ventricular ejection fraction (LVEF) and left ventricular internal dimension level. Blood urea nitrogen (BUN), serum creatinine (SCr), and urine (C035-2) were measured using enzyme-linked immunoassay (ELISA) kits (Nanjing Jiancheng Chemical Industrial Co., Ltd., Nanjing, China).

### Polymerase Chain Reaction (PCR)

Total ribonucleic acid (RNA) was extracted using Trizol Reagent (Life Sciences) and RNA was transcribed using the RevertAidTM First Strand cDNA Synthesis Kit (Life Sciences). PCR conditions were set as follows: 30 seconds at 95 °C for denaturation, 30 seconds at 55 °C for annealing, 30 seconds at 72 °C for extension. Forty cycles of amplifications were performed for each gene. Quantities of messenger ribonucleic acid (mRNA) were normalized to mRNA quantities of β-actin.

### Microarray Experiments

Microarray experiments were performed at the Genminix Informatics (China). Gene expression profiles were analyzed with the Human Exon 1.0 ST GeneChip (Affymetrix).

### Cell Culture and Transfection

Human umbilical vein endothelial cells were provided by Cell Bank (Shanghai, China) and cultured in Dulbecco’s Modified Eagle’s Medium (Gibco) containing 10% fetal bovine serum (Gibco) at 37 °C and 5% CO2. HSCARG plasmid, p47phox plasmid, nuclear factor kappa B (NF-κB) plasmid, siHSCARG mimics, sip47phox mimics, siNF-κB mimics, negative control plasmid, or negative mimics were purchased from Sangon Biotech Co., Ltd. (Shanghai, China). Cell was transfected with HSCARG plasmid (100 ng), p47phox plasmid (100 ng), NF-κB plasmid (100 ng), siHSCARG mimics (20 ng), sip47phox mimics (20 ng), siNF-κB mimics (20 ng), negative control plasmid (100 ng), or negative mimics (20 ng) using LipofectamineTM 2000 (Invitrogen, Carlsbad, California, United States of America). After 48 hours of transfection, cells were incubated with 10-6 M angiotensin II (Sigma, Shanghai, China).

### Western Blot

The total protein was extracted using radioimmunoprecipitation assay buffer (RIPA). 40 µg of protein samples were loaded separated on 10% sodium dodecyl sulphate-polyacrylamide gel electrophoresis (or SDS-PAGE) and transferred to nitrocellulose membranes (Millipore, United States of America). Membranes were blocked with 5% bovine serum albumin for 60 minutes and incubated with primary antibodies against HSCARG, p47phox, NF-κB, or β-actin overnight at 4 °C. After washing in the tris-buffered saline containing 0.1% Tween for 15 minutes, membranes were incubated with goat anti-rabbit or goat anti-mouse secondary antibodies for 1 hour at 37 °C. Membranes were visualized with enhanced chemiluminescence method by Tanon-5200 Chemiluminescence Imager (Tanon, Shanghai, China).

### Measurement of Oxidative Stress

Heart tissue samples were collected and homogenized using RIPA assay. Cell was collected at 1000 g for 10 minutes at 4 °C. Tissue samples or cell samples were used to measure ROS production, malondialdehyde (MDA), superoxide dismutase (SOD), glutathione (GSH), and glutathione peroxidase (GSH-PX) levels using corresponding ELISA kits.

### Statistical Analysis

*P*<0.05 was considered to indicate a statistically significant difference. Data were presented as mean ± standard deviation. All statistical analyses were carried out using IBM Corp. Released 2012, IBM SPSS Statistics for Windows, version 21.0, Armonk, NY: IBM Corp. Significance in two-condition experiments was evaluated by Student’s *t*-test or one-way analysis of variance (or ANOVA) test.

## RESULTS

### HSCARG Expression in Patients with Coronary Heart Disease or Mice Models

**Fig. 1:**
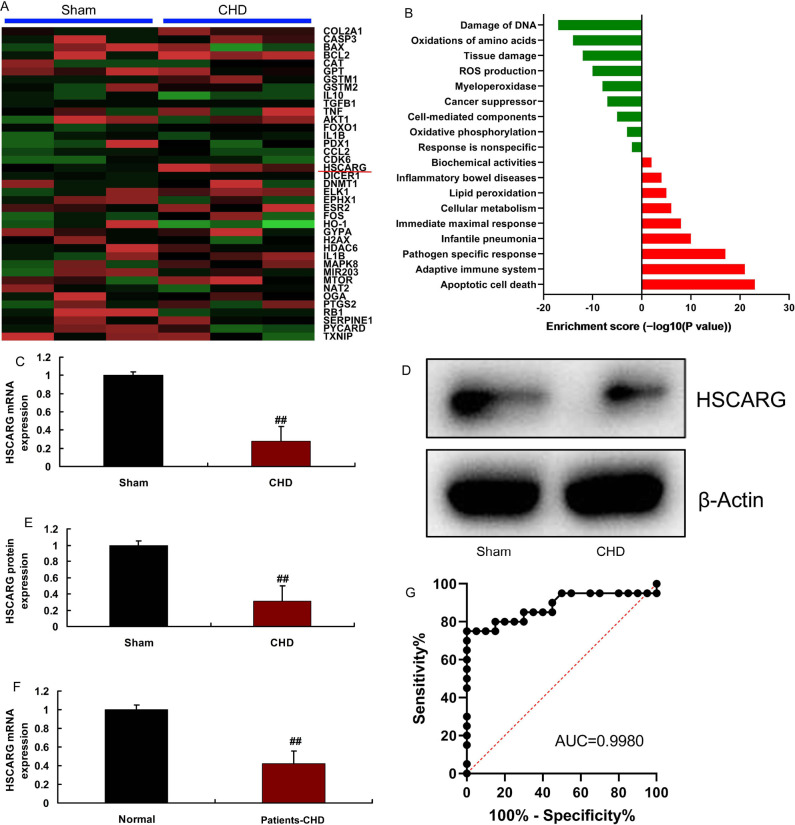
HSCARG expression in patients with atherosclerotic coronary heart disease (CHD) or mice model. Heat map and microarray data (A and B), HSCARG messenger ribonucleic acid (mRNA) and protein expression in mice model of CHD (C, D and E), serum HSCARG mRNA in patients with CHD (F), and sensitivity analysis (G) in patients with CHD. Sham=sham control group; CHD=mice model of CHD group; normal=normal volunteers group; patients-CHD=patients with CHD group. ##P<0.01 compared with sham control group or normal volunteers group. AUC=area under the curve; DNA=deoxyribonucleic acid; ROS=reactive oxygen species

To clarify the expression levels of HSCARG in CHD, we firstly analyzed HSCARG expression in the heart tissue of mice model of CHD using gene chip (Figures 1A and 1B). HSCARG mRNA and protein expressions were reduced in heart tissue of mice model of CHD, compared with the sham group (Figure 1C-1E). Meanwhile, we found that serum HSCARG mRNA expression was also inhibited in patients with CHD, compared with the normal group (Figure 1F). Area under the curve was 0.9980 and sensitivity was more reliable (Figure 1G).

### HSCARG Reduced Oxidative Stress in Mice Models of Coronary Heart Disease

**Fig. 2:**
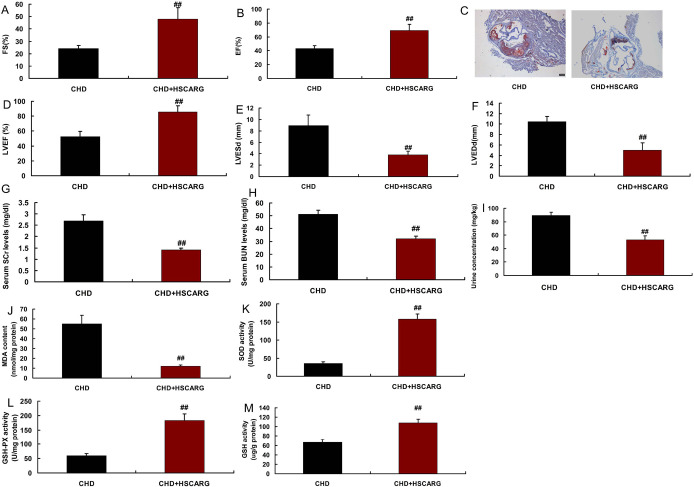
HSCARG reduced oxidative stress in mice model of atherosclerotic coronary heart disease (CHD). Fractional shortening (FS) (A), ejection fraction (EF) (B), plaque volume (hematoxylin and eosin staining) (C), left ventricular ejection fraction (LVEF) (D), left ventricular end-systolic diameter (LVESd) (E), left ventricular end-diastolic diameter (LVEDd) (F), serum creatinine (SCr) and blood urea nitrogen (BUN) (G and H), urine concentration (I), malondialdehyde (MDA) levels (J), superoxide dismutase (SOD), glutathione peroxidase (GSH-PX), and glutathione (GSH) levels (K, L, and M). CHD=mice model of CHD group; CHD+HSCARG=mice model of CHD treated by HSCARG protein group. ##P<0.01 compared with mice model of CHD group

Next, we used human HSCARG protein to weaken CHD. This study found that human HSCARG protein increased EF, FS, and LVEF levels, reduced plaque volume, LVEDd, and LVESd levels, inhibited SCr and BUN levels, decreased urine concentration and MDA levels, and promoted SOD, GSH, and GSH-PX levels in mice models of CHD (Figure 2).

### HSCARG Reduced ROS-Oxidative Stress in *in Vitro* Model

**Fig. 3:**
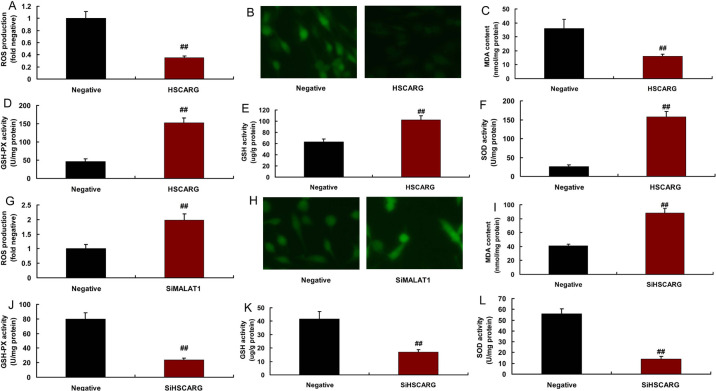
HSCARG reduced reactive oxygen species (ROS)-oxidative stress in in vitro model. ROS production levels (A and B), malondialdehyde (MDA), glutathione peroxidase (GSH-PX), glutathione (GSH), and superoxide dismutase (SOD) levels (C, D, E, and F) in in vitro model by overexpression of HSCARG; ROS production levels (G and H), MDA, GSH-PX, GSH, and SOD levels (I, J, K and L) in in vitro model by downregulation of HSCARG. Negative=negative mimic group; HSCARG=overexpression of HSCARG group; SiHSCARG=downregulation of HSCARG group. ##P<0.01 compared with negative mimic group

To analyze the effects and mechanism of HSCARG in CHD, we used *in vitro* model to confirm the antioxidant effects of HSCARG. Overexpression of HSCARG reduced ROS production and MDA levels, and increased SOD, GSH-PX, and GSH levels in the *in vitro* model (Figure 3A-3F). Downregulation of HSCARG promoted ROS production and MDA levels, and decreased SOD, GSH-PX, and GSH levels in *in vitro* models (Figure 3G-3L).

### HSCARG Suppressed p47phox Expression in *in Vitro* Model by NF-κB Activity

**Fig. 4:**
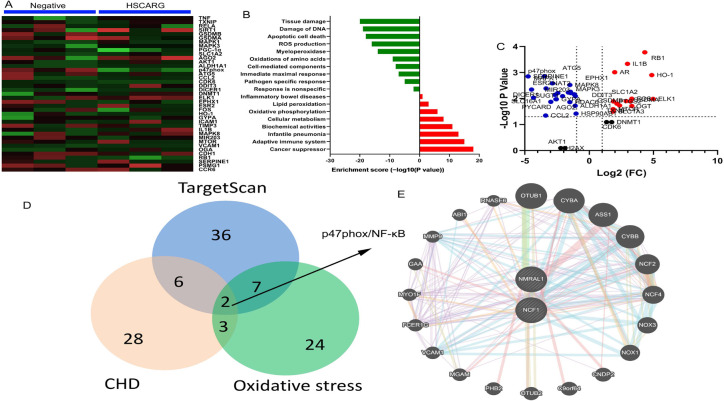
p47phox is an important target for HSCARG. Heat map and microarray data (A and B), volcanoes map (C), interpretation of result (D), network signal map (E). CHD=coronary heart disease; DNA=deoxyribonucleic acid; FC=fold change; NF-κB=nuclear factor kappa B; ROS=reactive oxygen species

**Fig. 5:**
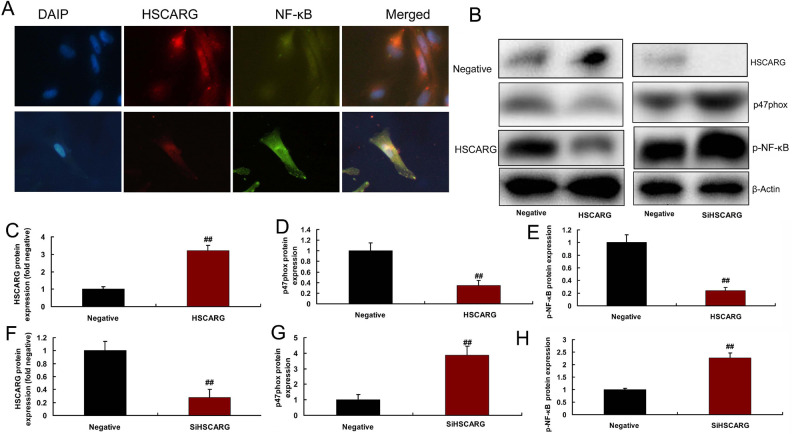
HSCARG suppressed p47phox expression in in vitro model by nuclear factor kappa B (NF-κB) activity. HSCARG and p47phox expression (immunofluorescence, A), HSCARG/47phox/NF-κB protein expressions by overexpression in in vitro model (B, C, D, and E); HSCARG/47phox/NF-κB protein expressions by downregulation in in vitro model (B, F, G, and H). Negative=negative mimic group; HSCARG=overexpression of HSCARG group; SiHSCARG=downregulation of HSCARG group. ##P<0.01 compared with negative mimic group. DAPI=2-(4-Amidinophenyl)-6-indolecarbamidine dihydrochloride

To analyze the mechanism of HSCARG in CHD, we used gene chip to measure the expression of gene in *in vitro* model. We found that overexpression of HSCARG reduced p47phox and p-NF-κB expression in *in vitro* model, which may be an important target (Figure 4). Immunofluorescence showed that HSCARG suppressed p47phox expression in *in vitro* model (Figure 5A). Overexpression of HSCARG induced HSCARG protein expression and suppressed the protein of p47phox and p-NF-κB expressions in *in vitro* model (Figure 5B-5E). And downregulation of HSCARG suppressed HSCARG protein expression and induced p47phox and NF-κB protein expressions in *in vitro* model (Figure 5B, 5F-5H).

### Regulation of NF-κB Activity in the Effects of HSCARG in Coronary Heart Disease

**Fig. 6:**
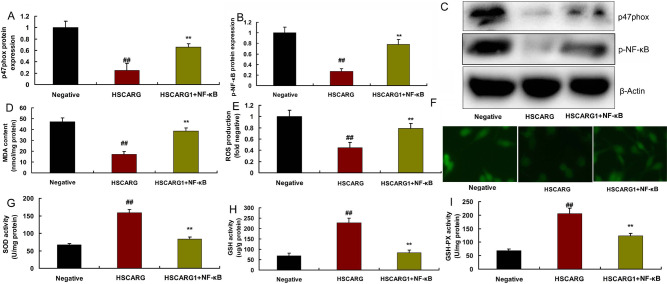
The overexpression of nuclear factor kappa B (NF-κB) activity in the effects of HSCARG in coronary heart disease. 47phox/NF-κB protein expressions (A, B, and C), malondialdehyde (MDA) and reactive oxygen species (ROS) production levels (D, E, and F), superoxide dismutase (SOD), glutathione (GSH), and glutathione peroxidase (GSH-PX) levels (G, H, and I). Negative=negative mimic group; HSCARG=overexpression of HSCARG group; HSCARG+NF-κB=overexpression of HSCARG and NF-κB group. ##P<0.01 compared with negative mimic group; **P<0.01 compared with overexpression of HSCARG group

**Fig. 7:**
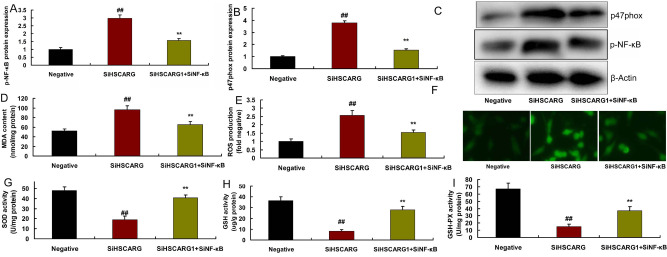
Downregulation of nuclear factor kappa B (NF-κB) activity in the effects of HSCARG in coronary heart disease. 47phox/NF-κB protein expressions (A, B, and C), malondialdehyde (MDA) and reactive oxygen species (ROS) production levels (D, E, and F), superoxide dismutase (SOD), glutathione (GSH), and glutathione peroxidase (GSH-PX) levels (G, H, and I). Negative=negative mimic group; SiHSCARG=downregulation of HSCARG group; SiHSCARG+siNF-κB=downregulation of HSCARG and NF-κB group. ##P<0.01 compared with negative mimic group; **P<0.01 compared with downregulation of HSCARG group

To detect the role of NF-κB activity in the effects of HSCARG in CHD, NF-κB plasmid and siNF-κB plasmid were used to regulate expression of NF-κB in *in vitro* model by regulation of HSCARG. NF-κB plasmid induced p-NF-κB and p47phox protein expressions, increased MDA and ROS production levels, and reduced SOD, GSH-PX, and GSH levels in *in vitro* model by overexpression of HSCARG (Figure 6). SiNF-κB plasmid suppressed p-NF-κB and p47phox protein expressions, decreased MDA and ROS production levels, and reduced SOD, GSH-PX, and GSH levels in *in vitro* model by overexpression of HSCARG (Figure 7).

### Regulation of p47phox in the Effects of HSCARG in Coronary Heart Disease

**Fig. 8:**
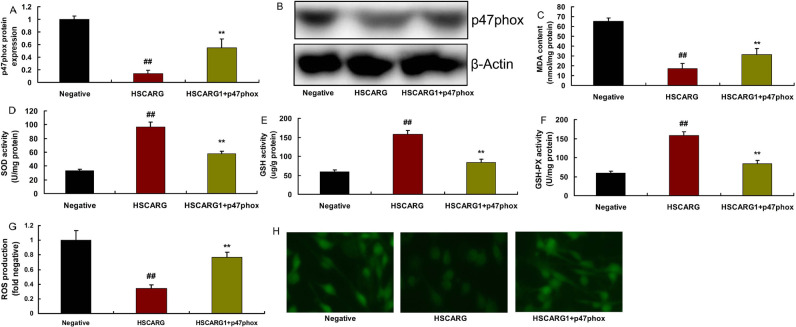
Overexpression of p47phox in the effects of HSCARG in coronary heart disease. 47phox protein expression (A and B), malondialdehyde (MDA) (C), superoxide dismutase (SOD), glutathione peroxidase (GSH-PX), and glutathione (GSH) levels (D, E, and F), reactive oxygen species (ROS) production levels (G and H). Negative=negative mimic group; HSCARG=overexpression of HSCARG group; HSCARG+p47phox=overexpression of HSCARG and p47phox group. ##P<0.01 compared with negative mimic group; **P<0.01 compared with overexpression of HSCARG group.

We further elucidated the relation between p47phox and HSCARG in CHD. p47phox plasmid induced p47phox protein expression, increased MDA levels and ROS production levels, and reduced SOD, GSH-PX, and GSH levels in *in vitro* model by overexpression of HSCARG (Figure 8).

## DISCUSSION

Atherosclerotic CHD is also known as atherosclerotic ischemic heart disease^[15]^. CHD is caused by coronary AS, which leads to stenosis and occlusion of the lumen^[16]^. Occasionally, coronary artery spasm, disturbance of coronary microcirculation, myocardial metabolic abnormalities, etc., can cause myocardial ischemia and hypoxia, thereby leading to CHD^[16,17]^. In the present study, HSCARG expression was inhibited in patients or mice with CHD. Zhao et al.^[18]^ indicated that HSCARG downregulation is essential for epithelial cell viability. So, HSCARG may be a regulating factor for CHD.

Oxidative stress is one of the important causes of abnormal cardiovascular structure and function, which also plays an important role in the occurrence and development of CHD^[19]^. It not only promotes the oxidative modification and lipid peroxidation of low-density lipoprotein in CHD, but also induces changes in vascular gene expression and promotes cell proliferation^[20-22]^. In the present study, HSCARG reduced ROS-induced oxidative stress in mice and *in vitro* model of CHD. Zang et al.^[14]^ expanded that HSCARG negatively regulates the translesion synthesis pathway and reduced oxidative stress. This finding suggests that HSCARG reduced ROS-induced oxidative stress to prevent CHD.

NOX is the main source of reactive oxygen in blood vessels, which has also been confirmed as an important source of reactive oxygen in the progress of CHD. p47phox regulates the activation of NOX, thereby generating a large amount of ROS^[23]^. The excessive ROS over scavenging capacity of the body can activate intracellular signaling pathways by increasing calcium ion levels, changing the redox state of cells, and upregulating the expression of inflammatory molecules, promoting smooth muscle cell proliferation and oxidative modification of low-density lipoprotein, causing changes in vasoconstriction, ultimately leading to the occurrence and development of CHD^[24,25]^. Our results suggested that HSCARG suppressed p47phox expression in *in vitro* model by NF-κB activity. Therefore, it is worthy to elucidate HSCARG suppression of NF-κB/p47phox to reduce ROS-induced oxidative stress in CHD.

### Limitations

This paper only used one cell line (human umbilical vein endothelial cells) for the *in vitro* model, which was one limitation of this study.

## CONCLUSION

In conclusion, we demonstrate that HSCARG expression was inhibited in patients or mice with CHD. HSCARG regulated NF-κB/p47phox passage to reduce ROS-induced oxidative stress in CHD, which further elucidated the detailed mechanism of how HSCARG inhibits NF-κB activity in CHD. The results of the present study suggest that HSCARG may be a clinical target and a potential therapeutic treatment for CHD in clinical scenarios.
